# Frequency and Diversity of Hybrid *Escherichia coli* Strains Isolated from Urinary Tract Infections

**DOI:** 10.3390/microorganisms9040693

**Published:** 2021-03-27

**Authors:** Júllia A. S. Nascimento, Fernanda F. Santos, Tiago B. Valiatti, José F. Santos-Neto, Ana Carolina M. Santos, Rodrigo Cayô, Ana C. Gales, Tânia A. T. Gomes

**Affiliations:** 1 Laboratório Experimental de Patogenicidade de Enterobactérias (LEPE), Disciplina de Microbiologia, Departamento de Microbiologia, Imunologia e Parasitologia (DMIP), Escola Paulista de Medicina (EPM), Universidade Federal de São Paulo (UNIFESP), São Paulo 04023-062, Brazil; jullia.nascimento@unifesp.br (J.A.S.N.); ff.santos@unifesp.br (F.F.S.); tiago.valiatti@unifesp.br (T.B.V.); jfs.neto@unifesp.br (J.F.S.-N.); carolina.mello@unifesp.br (A.C.M.S.); 2 Laboratório Alerta, Disciplina de Infectologia, Departamento de Medicina, Escola Paulista de Medicina (EPM), Universidade Federal de São Paulo (UNIFESP), São Paulo 04039-032, Brazil; rodrigo.silva@unifesp.br (R.C.); ana.gales@unifesp.br (A.C.G.); 3 Laboratório de Imunologia e Microbiologia (LIB), Setor de Biologia Molecular, Microbiologia e Imunologia, Departamento de Ciências Biológicas (DCB), Instituto de Ciências Ambientais, Químicas e Farmacêuticas (ICAQF), Universidade Federal de São Paulo (UNIFESP), Diadema 09972-270, Brazil

**Keywords:** urinary tract infection, hybrid strains, UPEC, DEC virulence markers, antimicrobial resistance

## Abstract

(1) Background: Hybrid uropathogenic *Escherichia coli* (UPEC) strains carry virulence markers of the diarrheagenic *E. coli* (DEC) pathotypes, which may increase their virulence potential. This study analyzed the frequency and virulence potential of hybrid strains among 452 UPEC strains. (2) Methods: Strains were tested for the DEC virulence diagnostic genes’ presence by polymerase chain reaction (PCR). Those carrying at least one gene were classified as hybrid and further tested for 10 UPEC and extraintestinal pathogenic *E. coli* (ExPEC) virulence genes and phylogenetic classification. Also, their ability to produce hemolysis, adhere to HeLa and renal HEK 293T cells, form a biofilm, and antimicrobial susceptibility were evaluated. (3) Results: Nine (2%) hybrid strains were detected; seven of them carried *aggR* and two, *eae*, and were classified as UPEC/EAEC (enteroaggregative *E. coli*) and UPEC/aEPEC (atypical enteropathogenic *E. coli*), respectively. They belonged to phylogroups A (five strains), B1 (three), and D (one), and adhered to both cell lineages tested. Only the UPEC/EAEC strains were hemolytic (five strains) and produced biofilm. One UPEC/aEPEC strain was resistant to third-generation cephalosporins and carried *bla*_CTX-M-15_. (4) Conclusions: Our findings contribute to understanding the occurrence and pathogenicity of hybrid UPEC strains, which may cause more severe infections.

## 1. Introduction

*Escherichia coli* is a commensal microorganism of the gastrointestinal tract of mammals. However, some *E. coli* strains can be considered pathogenic due to the acquisition of different virulence-encoding genes during their evolution, which allowed them to cause intestinal or extraintestinal infections [1]. According to the body site of infection and their virulence markers, these pathogens can be divided into two different groups called diarrheagenic (DEC) and extraintestinal pathogenic (ExPEC) *E. coli*. The DEC group comprises six pathotypes: enteropathogenic *E. coli* (EPEC), Shiga toxin-producing *E. coli* (STEC) (including the enterohemorrhagic *E. coli* strains (EHEC)), enterotoxigenic *E. coli* (ETEC), enteroaggregative *E. coli* (EAEC), enteroinvasive *E. coli* (EIEC), and diffusely adherent *E. coli* (DAEC) [2]. Four pathotypes compose the ExPEC group: uropathogenic *E. coli* (UPEC), neonatal meningitis-associated *E. coli* (NMEC), human sepsis-associated *E. coli* (SEPEC), and avian pathogenic *E. coli* (APEC) [3,4]. Unlike DEC, the ExPEC pathotypes were defined by isolation source, share different sets of virulence factors (VFs), and pathogenic strains to humans are pathogenic to animals and vice-versa [5,6]. To date, more than 50 VFs were described as playing a role in ExPEC pathogenesis [7,8]. All this diversity enables ExPEC strains to cause different types of infections and diseases, like urinary tract infections (UTIs), bloodstream infections, and meningitis, besides having been associated with prostate infections and colorectal cancer development [5,6,9,10].

UPEC is the most common ExPEC pathotype and is considered the leading cause of community-acquired urinary tract infections (UTIs) and most healthcare-associated infections of the urinary tract [11,12,13,14,15]. UPEC strains have a significant genetic diversity that contributes to colonization and persistence in the urinary tract, even in immunocompetent patients. Despite the numerous VFs related to the occurrence of UTIs, including important adhesins like P and type 1 fimbriae and iron acquisition systems, like yersiniabactin, studies evaluating lethality in a mice subcutaneous sepsis model showed a correlation between the presence of specific VFs and the capacity of *E. coli* strains to cause infections in immunocompetent subjects [16,17,18]. Therefore, the presence of at least two of five genes (*pap*, *afa/dra*, *sfa*, *kpsMTII*, and *iut/iuc*) identifies ExPEC strains that bear intrinsic virulence and can cause any extraintestinal infection in immunocompetent individuals [16]. Later, another study focused on evaluating the capacity of strains exclusively to cause UTIs, considering that some strains could cause only UTIs and not systemic infections. Using the ascendent urinary tract infection model in mice and evaluating a large set of VFs, they identified that *E. coli* strains that bear *chuA, fyuA, vat*, and *yfcV* simultaneously can cause UTIs, regardless of the host conditions [19]. Additionally, some pathogenic strains bear only ExPEC VFs, and others, only UPEC VFs [19]. Thus, these VFs can be used as molecular markers to identify and track ExPEC strains regardless of the isolation source or host conditions, and could be used complementarily [16,19].

Although horizontal gene transfer (HGT) is associated with the evolution and genomic complexity of UPEC strains, such events can also contribute to the emergence of hybrid strains, that is, strains isolated from the urinary tract that exhibit DEC defining virulence genes [20]. Hybrid strains may carry VFs from intestinal and extraintestinal pathotypes, which may contribute to the increase of strains pathogenicity [21]. However, the frequency of hybrid UPEC strains in the clinical setting remains to be defined. This study aimed to analyze the frequency of hybrid UPEC strains among *E. coli* strains recovered from patients with UTIs and characterize molecularly and phenotypically their virulence background.

## 2. Materials and Methods

### 2.1. Samples and Strains Identification

The study comprised 452 non-duplicate *E. coli* isolates recovered between August 2018 and March 2019 from adult patients with UTIs. The urine samples were collected in the Central Laboratory of Hospital São Paulo (HSP), which handles clinical samples of inpatients and ambulatory patients from three hospitals of the HSP complex, located in São Paulo city, Brazil. The isolates were stored at −80 °C in cryotubes containing Lysogeny Broth (LB) (ThermoFisher Scientific, Basingstoke, UK) and 15% glycerol.

The isolates were obtained from 341 female patients (mean age of 47 years old, ranging from 1 to 95 years old) and 87 male patients (mean age of 48 years old, ranging from 1 to 85 years old) with UTIs attended in the HSP complex. Information on the gender and age of the remaining 24 strains was unavailable. The anonymized medical records of patients carrying the hybrid strains were evaluated to identify the type of infection and any host medical condition. The present study was performed with the approval of the local Research Ethics Committee of the Federal University of São Paulo—UNIFESP/São Paulo Hospital (CEP number 3996160919 of October 2019).

The identification of all 452 strains was performed using the Matrix-Assisted Laser Desorption Ionization-Time of Flight Mass Spectrometry (MALDI-TOF MS) technique on the Microflex LT (BrukerDaltonics, Billerica, MA, USA) equipment. The bacterial colonies obtained from overnight cultures were homogenized in 900 µL of ethanol (100%) and centrifuged at 8000× *g* for 2 min. Afterward, the supernatant was discarded, and 50 µL of formic acid (70%) and 50 µL of acetonitrile were added for the complete dissolution of the pellet. The solution was subsequently centrifuged again at 8000× *g* for 2 min, and 1 µL of the supernatant was transferred to a MALDI-TOF MS plate. After drying at room temperature, the supernatant was covered with 1 µL of matrix solution (α-cyano-4-hydroxycinnamic acid). After drying once again, the plate was placed into the equipment for MALDI-TOF MS analyzes. Results were analyzed with MALDI biotyper software version 3.3 (BrukerDaltonics, Billerica, MA, USA) using the following cut-off values: ≥1.7–1.99 for the genus identification and ≥2.0–2.99 for species identification. Scores < 1.7 were considered unreliable.

### 2.2. Molecular Characterization of Hybrid Strains

Total bacterial DNA for each strain was obtained by the thermal lysis method [22]. Subsequently, the presence of DEC virulence-encoding genes was screened by polymerase chain reaction (PCR) using specific primers, as shown in Table S1. Those strains that presented at least one of the DEC pathotypes’ diagnostic markers were considered hybrid strains (Table S1). The detection of at least two of five specific virulence genes (*papC*, *sfaDE*, *afaBCIII*, *kpsMTII,* and *iucD*) characterized the strains with intrinsic virulence [16]. The uropathogenic potential of the strains was assessed using the methodology of Spurbeck et al. (2012), in which the simultaneous presence of the *chuA*, *vat*, *fyuA*, and *yfcV* markers suggests a correlation with a uropathogenic potential [19]. The hybrid strains were also classified phylogenetically, according to the criteria established by Clermont et al. (2013), using the quadruplex PCR technique for the following genes: *chuA*, *yjaA*, TspE4.C2, and *arpA*, followed by duplex PCR to classify the different phylogenetic groups [23]. The *hlyA* and *ehx* genes’ detection was performed using the PCR methodology described previously [24].

### 2.3. Phenotypic Detection of Hemolytic Activity

The hybrid strains were initially grown in LB overnight to detect the production of hemolysins. After that, 10 µL of a bacterial suspension adjusted to 10^6^ CFU/mL were inoculated on top of tryptic soy agar plates (Difco Laboratories, Detroit, MI, USA) supplemented with 10 mM CaCl_2_ and 5% of defibrinated sheep blood (Laborclin^®^, São Paulo, SP, Brazil) previously washed with phosphate-buffered saline (PBS). The occurrence of hemolysis was observed after 3 h, 6 h, and 18 h of incubation at 37 °C [25]. The *E. coli* strains J96 and EDL933, producing α hemolysin and enterohemolysin, respectively, were used as controls for phenotypic detection of hemolytic activity. The *E. coli* strain C600 was used as a non-hemolytic control.

### 2.4. Cell Culture and Maintenance

HeLa (ATCC^®^ CCL-2™) and HEK 293T (ATCC^®^ CRL-11268) cell lineages were used to evaluate the ability of hybrid strains to interact with eukaryotic epithelial cells. Both lineages were cultured in Dulbecco’s Modified Eagle Medium (DMEM), high glucose, GlutaMax™ (Gibco-ThermoFisher Scientific, Grand Island, NY, USA), containing 15 mM HEPES (Sigma-Aldrich, Sant Louis, MO, USA), supplemented with 10% bovine fetal serum (BFS) (Gibco-ThermoFisher Scientific), 1% nonessential amino acids (Gibco, Life Technologies, Carlsbad, CA, USA), and 1x antibiotic mixture (penicillin—5 mg/mL, streptomycin—5 mg/mL; neomycin—10 mg/mL) (PSN) (Gibco, Life Technologies). The lineages were kept at 37 °C in an atmosphere of 5% CO_2_. For all assays, cell suspensions containing 1 × 10^5^ cells/mL were seeded in 24-well microplates containing 13 mm diameter glass coverslips and cultured for 48 h to obtain ~80% confluence.

### 2.5. Adherence Assay

The hybrid strains’ adherence capacity was assessed following the protocol used in our laboratory [26]. The cells prepared as described above were washed three times with PBS (pH 7.4) and further cultivated with 1 mL of the same medium, except that no antibiotics were added and BFS was used at a concentration of 2%. In the experiments with HeLa cells, 2% methyl-D-mannose (Sigma-Aldrich) was added to the medium to prevent type 1 fimbriae-mediated bacterial adherence, allowing adherence pattern identification [26]. Cells were infected with 20 µL of a bacterial suspension obtained from cultures grown overnight in LB (∼10^8^ CFU/mL), generating a multiplicity of infection (MOI) of 10. After 3 h or 6 h of incubation at 37 °C in a normal atmosphere, the microplates were washed three times with PBS, and coverslips were fixed with methanol at room temperature for 30 min, stained with May-Grünwald/Giemsa (Merck, Darmstadt, Germany), and analyzed under immersion light microscopy. As controls of adherence patterns, prototype strains producing aggregative adherence (AA; EAEC 042), localized adherence (LA; typical EPEC E2348/69), and localized adherence-like (LAL; aEPEC 4581-2) were used. The CFT073 strain was used as a UPEC control, and non-adherent *E. coli* strain HB101 and non-infected cells were used as negative controls.

### 2.6. Biofilm Formation Assay

The biofilm formation assay was performed using 96-well polystyrene plates, as previously published with minor modifications [27]. Strains were grown in LB and incubated at 37 °C for 18 h. After that, 5 µL of each overnight culture were added into 200 µL of LB or DMEM GlutaMAX (ThermoFisher Scientific, Carlsbad, CA, USA) and incubated at 37 °C for 24 h. Subsequently, successive gentle washes with PBS were performed, and preparations were fixed in 3% formaldehyde and stained with 1 mL of 0.5% crystal violet. The optical density reading was performed in a spectrophotometer (EnSpire Multimode Plate Reader, PerkinElmer, Walthman, MA, USA) at 620 nm after the dye solubilization with 95% ethanol (200 µL/well). The results were obtained from the average of an experimental triplicate. EAEC 042 and laboratory *E. coli* HB101 strains were used as positive and negative controls in all assays, respectively. The prototype strain CFT073 was used as a UPEC control, and a non-infected well was used as a control of dye retention.

### 2.7. Antimicrobial Susceptibility Testing

The Minimal Inhibitory Concentrations (MICs) for ampicillin/sulbactam, ceftriaxone, ceftazidime, cefepime, aztreonam, ertapenem, imipenem, meropenem, ciprofloxacin, levofloxacin, amikacin, gentamicin, and minocycline (Sigma, Saint Louis, MO, USA) were determined by agar dilution, except for polymyxins and tigecycline, where the cation-adjusted broth microdilution method was performed [28]. The results were interpreted according to the Brazilian Committee on Antimicrobial Susceptibility Testing (BrCAST/EUCAST) guidelines using the breakpoint Table version 10.0, published in may 20, 2020 (http://brcast.org.br/ (accessed on 1 January 2021)). *E. coli* ATCC 25922 and *Pseudomonas aeruginosa* ATCC 27853 were used as control strains.

### 2.8. Detection of EsβL Encoding Genes

The strains that showed ESβL phenotype through the double-disc synergy test were subjected to the screening for the main ESβL encoding genes: *bla*_CTX-M1/2_, *bla*_CTX-8_, *bla*_CTX-14_, *bla*_TEM_, *bla*_GES_, and *bla*_SHV_, by PCR. To identify the gene variant when positive, amplicons were sequenced [29].

### 2.9. Ethics Approval

Ethical approval for this study was obtained from the Research Ethics Committee from the Federal University of São Paulo—UNIFESP/São Paulo Hospital (Process number: 3996160919).

### 2.10. Statistical Analyses

The One-way ANOVA followed by post-hoc Tukey HSD test was used to compare the results using the threshold for statistical significance as *p*-value ≤ 0.05. The analyses were performed in Prism 5.0 (GraphPad Prism Software, Inc., San Diego, CA, USA).

## 3. Results

### 3.1. Characterization of Hybrid UPEC Strains

Among the 452 UPEC strains evaluated, nine (2%) presented at least one of the key genes used to define the DEC pathotypes, being considered hybrid strains. Seven of them carried the *aggR* gene, which defines the EAEC pathotype, and in this study were classified as hybrid UPEC/EAEC (Table 1). The other two strains harbored *eae* but not *bfpB* or *stx* and were thus considered hybrid UPEC/aEPEC (atypical EPEC) (Table 1). The presence of *eae* in isolates devoid of *bfpB* and *stx* is the diagnostic marker of aEPEC. No ETEC, EIEC, or STEC virulence markers were identified among the 452 UPEC strains.

Seven of the hybrid strains were isolated from patients from the community seeking ambulatory hospital care with symptoms related to cystitis (six cases) and pyelonephritis (one case). Two of them, HSP 199 and HSP 215, were isolated from patients with pyelonephritis and recurrent cases of cystitis, respectively (Table 2).

Among the UPEC/EAEC strains, three of them carried the intrinsic virulence markers of ExPEC: *afaBC*, *iucD,* and *kpsMTII* besides *fyuA* (a UPEC marker). Another two strains carried *papC* and *fyuA,* and one carried *chuA*. The latter did not carry any of the ExPEC or UPEC defining VFs (Table 1). Concerning the uropathogenic potential, the *fyuA* and *chuA* genes were detected, respectively, in six and one of the hybrid strains.

The phylogenetic origin of the UPEC/EAEC strains was diverse since the majority belonged to phylogroup A (*n* = 4), followed by phylogroup B1 (*n* = 2), and phylogroup D (*n* = 1) (Table 1). One of the UPEC/aEPEC strains carried the *iucD* gene and harbored *fyuA* (Table 1), which is essential for iron uptake in *E. coli*. The two UPEC/aEPEC strains belonged to different phylogroups (A and B1) (Table 1).

### 3.2. Virulence Potential of Hybrid UPEC Strains

**Figure 1:**
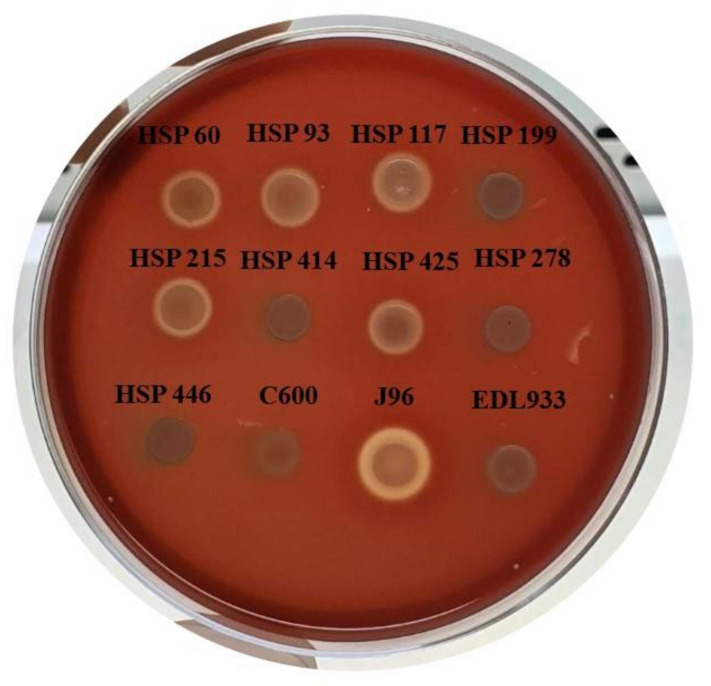
Phenotype of hemolytic activity of hybrid *Escherichia coli* strains isolated from urinary tract infection on tryptic soy broth agar supplemented with CaCl_2_ and washed defibrinated sheep blood after 24 h of incubation at 37 °C. *E. coli* strains used as controls: EDL933 (enterohemolysin-producer), J96 (alpha-hemolysin producer), and C600 (nonhemolytic).

Concerning the hemolytic phenotype, four of nine hybrid strains produced clear hemolysis after 3 h (HSP 60, HSP 93, HSP 117, and HSP 215) and one after 6 h (HSP 425) of incubation. Four strains did not show hemolytic activity up to 24 h, as shown in Table 1 and Figure 1. PCR analyses confirmed the presence of the α-hemolysin-encoding gene, *hlyA,* for all hemolytic strains.

**Figure 2:**
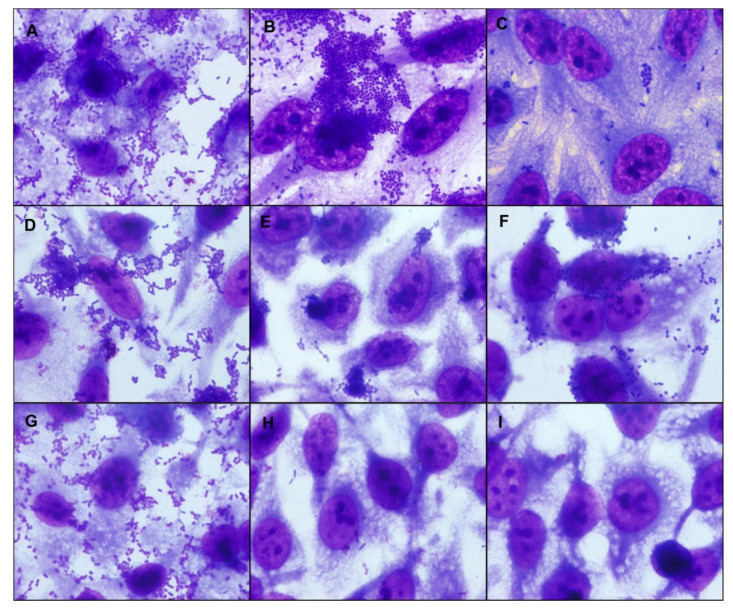
Adherence patterns of representative hybrid uropathogenic *Escherichia coli* (UPEC) strains. The adherence patterns were assessed as preconized in HeLa cells in assays with an incubation period of 3 h or 6 h, at 37 °C in the presence of 2% D-mannose, using a multiplicity of infection of 10. The preparations were stained with May-Grünwald/Giemsa and observed under a light optical microscope (1000× magnification). All hybrid UPEC strains were adherent, and different adherence patterns were identified. Representative hybrid UPEC/EAEC (enteroaggregative *E. coli*) strains (3 h) are in panels (**A**,**B**), and a hybrid UPEC/aEPEC (atypical enteropathogenic *E. coli*) strain (6 h) in panel (**C**). (**A**). HSP 117, displaying a non-characteristic aggregative adherence pattern with small loose clusters and spread foci of adherent bacteria; (**B**). HSP 414, showing the typical aggregative adherence pattern; (**C**). HSP 278, showing the localized adherence-like pattern. (**D**). *E. coli* 042 (EAEC—aggregative adherence pattern control); (**E**). *E. coli* strain E2348/69 (typical EPEC—localized adherence pattern control); (**F**). *E. coli* strain 4581-2 (aEPEC—localized adherence-like pattern control); (**G**). *E. coli* strain CFT073 (UPEC control); (**H**). *E. coli* strain HB101 (K-12 derived laboratory strain, non-adherent control); (**I**). Non-infected control cells.

**Figure 3:**
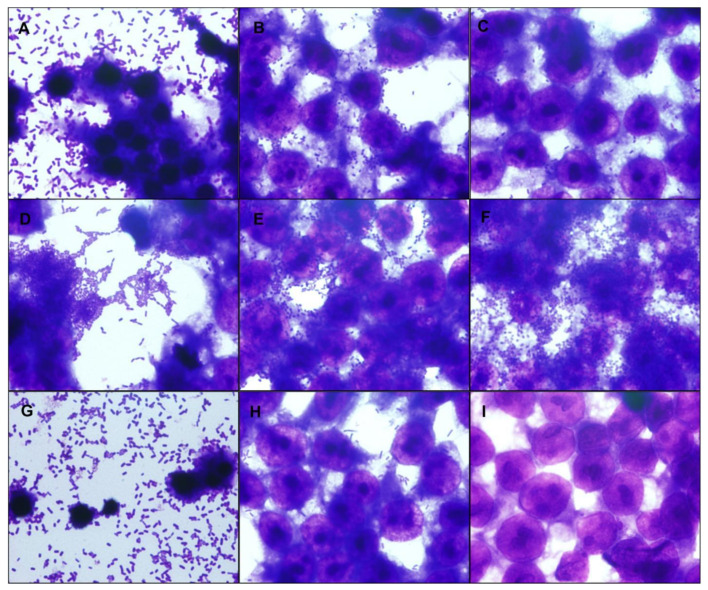
Interaction with a renal origin cell-lineage. The hybrid uropathogenic *Escherichia coli* (UPEC) strains’ capacity to interact with human renal cells was assessed using HEK 293T cells in assays with an incubation period of 3 h, at 37 °C without D-mannose, using a multiplicity of infection of 10. The preparations were stained with May-Grünwald/Giemsa and observed under a light optical microscope (1000× magnification). Representative hybrid UPEC/EAEC (enteroaggregative *E. coli*) strains are in panels (**A**,**B**), and a hybrid UPEC/aEPEC (atypical enteropathogenic *E. coli*) strain in panel (**C**). All hybrid UPEC strains interacted with renal cells in diverse intensity; in panel (**A**), the HEK 293T cell monolayer was partially detached, and pyknotic nuclei were observed in the remaining cells; the same phenotype was observed in panel (**G**), with CFT073, a UPEC control strain, which produces hemolysin. (**A**) HSP 117; (**B**) HSP 414; (**C**) HSP 278; (**D**) *E. coli* strain 042 (EAEC control); (**E**) *E. coli* strain E2348/69 (typical EPEC control); (**F**) *E. coli* strain 4581-2 (aEPEC control); (**G**) *E. coli* strain CFT073 (UPEC control); (**H**) *E. coli* strain HB101 (*E. coli* K-12 derived laboratory strain, non-adherent control); (**I**) Non-infected control cells.

The adherence assays on HeLa cells were carried out in 3 h and 6 h for UPEC/EAEC and UPEC/aEPEC strains, respectively, to determine their adherence patterns. Five of seven UPEC/EAEC strains produced a non-characteristic aggregative adherence pattern (Figure 2 and Figure S1). These results showed that such UPEC/EAEC strains did not present the typical standard stacked brick pattern used to identify EAEC strains. Two UPEC/EAEC strains presented the typical EAEC pattern produced by the prototype EAEC 042 strain. The two UPEC/aEPEC strains formed small loose clusters, characteristic of aEPEC (Figure 2 and Figure S1). The renal lineage HEK 293T cell assay was performed to determine the ability of hybrid UPEC strains to adhere to urinary tract cells. The UPEC/EAEC strains showed strong adherence to HEK 293T cells, especially strains HSP 117, HSP 215, and HSP 425 (Figure 3 and Figure S2). The adherence of UPEC/aEPEC strains in this lineage was less evident compared to UPEC/EAEC (Figure 3 and Figure S2).

**Figure 4:**
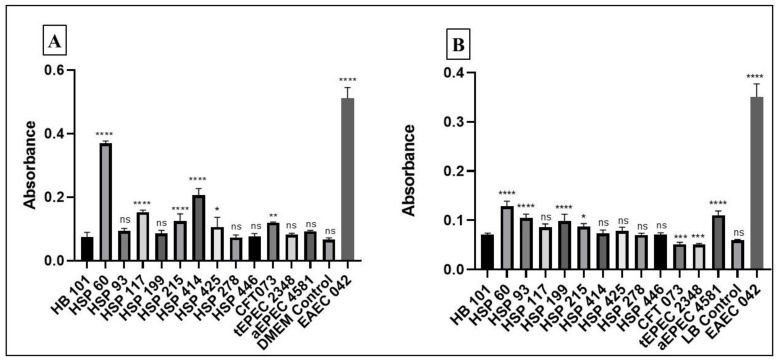
Biofilm formation with incubation period of 24 h, in Dulbecco Modified Essential Medium (DMEM) + GlutaMAX medium (**A**) and Lysogeny-Broth (LB) (**B**). Positive control: EAEC (enteroaggregative *E. coli*) prototype strain 042; Negative control: non-adherent *E. coli* strain HB101. The absorbance reading was performed at 620 nm. The One-way ANOVA followed by post-hoc Tukey HSD test was used to compare the results. *p* values: * *<* 0.05; ** *<* 0.01; *** *<* 0.001; **** *p* < 0.0001; ns > 0.05. The results were obtained from the average of a biological triplicate.

The biofilm formation assay demonstrated that five and four UPEC/EAEC strains produced biofilm in DMEM and LB media, respectively (Table 1). Strains HSP 60 and HSP 215 produced biofilm in both media (Figure 4). Furthermore, four of seven UPEC/EAEC strains (HSP 60, HSP 117, HSP 215, and HSP 414) produced strong biofilm in DMEM (Figure 4A), and three (HSP 60, HSP 93, and HSP 199) in LB (Figure 4B). No biofilm formation was observed among the UPEC/aEPEC strains (Table 1, Figure 4).

Regarding antimicrobial resistance, one of the strains (HSP 446) showed high MICs for ceftriaxone, ceftazidime, and cefepime (Table 3), a phenotype characteristic of ESBL producing strains. The amplicon sequencing revealed that UPEC HSP 446 carried the *bla*_CTX-M-15_ gene.

## 4. Discussion

Studies focusing on the genetic diversity of *E. coli* confirm that this species is continually evolving, especially by genome deletions and horizontal gene transfer processes [30]. Such genomic flexibility contributes to more significant intra-species variability [31], allowing the emergence of strains with enhanced pathogenic potential. Indeed, the term hybrid-pathogenic *E. coli* has been created to depict the emergence of strains carrying new combinations of DEC and ExPEC VFs or strains recovered from extraintestinal infections that carried DEC VFs [32]. Overall, the frequency of hybrid *E. coli* strains recovered from UTIs in the current study during seven consecutive months was 2% (9/452 strains). Seven of them were UPEC/EAEC hybrid strains since they carried the EAEC transcriptional activator of aggregative adherence (*aggR*) diagnostic marker, which encodes a global regulator of EAEC virulence genes [33]. Two strains were classified as UPEC/aEPEC hybrid strains because they were devoid of the *bfpB* and *stx* genes and harbored the *eae* gene that encodes an outer membrane adhesive protein (intimin), which is essential for the establishment of attaching and effacing lesions (AE) in the intestinal epithelium by EPEC and EHEC strains [20].

Despite the low occurrence, the detection of virulence genes typical of the DEC pathotypes among UPEC strains has been reported by a few studies [33,34,35,36]. In a previous study of our group [36], 3% (*n* = 7/225) of the UPEC strains evaluated carried the *aggR* gene, and one strain harbored the *eae* EPEC marker [36]. Interestingly, in that study, the strains were isolated at the same hospital complex of the current study (Hospital São Paulo) more than 20 years ago. Later, another study found that 28/265 (10.6%) of UPEC strains evaluated harbored at least one DEC virulence gene, being the *aggR* and *eae* genes detected in one and two strains, respectively [34]. In contrast, Lara et al. showed that 3.4% (9/258) of the UPEC strains studied carried *aggR* [33]. These studies showed that the frequency of hybrid *E. coli* strains varied according to the population examined [30,31,32] and corroborated our findings. Another significant hybrid strain to appraise is the STEC O2:H6 strain that showed both STEC and UPEC characteristics, being able to cause diarrhea and UTIs in the same patient [37]. Interestingly, in the present study, we did not identify any STEC hybrid strain. Altogether, our data and the literature reveal that it is essential to monitor the different aspects of hybrid strains for epidemiological purposes, considering their higher pathogenic potential, which can help in better management in outbreak situations and epidemiological knowledge.

Due to the scarce information on clinical and epidemiological aspects of patients infected by hybrid strains, more studies are necessary to understand their distribution and impact on affected patients’ clinical outcomes. Herein, we found that seven of the hybrid strains were related to cystitis or pyelonephritis; two (UPEC/EAEC) were isolated from patients with recurrent UTIs. However, it was not possible to determine if these hybrid strains were responsible for the disease recurrence. Also, the hybrid pathogenic *E. coli* strains were the only pathogen isolated from symptomatic patients’ urine. Moderate to high pain and hematuria were frequent findings in all patients with UTIs caused by hybrid strains. Additionally, most of these strains were community-acquired.

The isolation of the HSP 278 strain, which harbored only the DEC VFs, from a young adult without any medical conditions was suggestive of the capacity of this hybrid strain to cause extraintestinal infection, despite the lack of the most common VFs associated with UTIs, like P fimbriae, and sialic-acid capsule. Therefore, additional studies are required to unveil the mechanism involved in the establishment of these infections. Concerning the UPEC/EAEC strains, some studies had pointed out the involvement of aggregative adherence fimbriae (AFF) and other EAEC VFs with the occurrence of extraintestinal infections [38,39] and the importance of yersiniabactin in the development of extraintestinal infections [40], while is unclear whether any of the aEPEC VFs may play a role in extraintestinal infection.

Alpha-hemolysin (HlyA), encoded by the *hlyA* gene in a pathogenicity island, causes the lysis of erythrocytes, endothelial cells, and urinary tract cells, enabling bacteria to capture iron and escape from phagocytes [41,42]. This iron uptake process is essential for the UPEC persistence in the host, proliferation, and pathogenicity [43]. Previous studies also demonstrated that HlyA production might be related to severe infections, such as sepsis and kidney injury [37]. The study conducted by Firoozeh et al. demonstrated that HlyA production in UPEC strains causing pyelonephritis was more prevalent than in those strains causing cystitis, which also indicates the association of HlyA with severe infections [44]. Four UPEC/EAEC strains in the present study showed a characteristic hemolytic phenotype at 3 h of incubation and one (HSP 425) at 6 h and all carried *hlyA*, suggesting that these strains are highly virulent.

Interestingly, we also verified that the interaction of the HSP 60, HSP 93, HSP 117, and HSP 215 strains with HEK 293T caused cells to detach from coverslips with shrinkage of the remaining cells after 3 h. Such phenomenon was not observed with the HSP 425 strain, whose hemolytic activity was detected a little later (6 h), indicating that it produces or secrets less hemolysin. Previous studies [45,46,47] suggested that the detachment caused by certain *E. coli* strains in cell lineages could be due to HlyA production. The reason cell detachment and shrinkage by the hemolytic strains were not observed in HeLa cells remains to be studied.

Three previous studies showed the association of HlyA and the adhesin-encoding *pap* and *sfa* genes [48,49,50]. However, in our study, one of the HlyA-producing strains (HSP 215) did not simultaneously carry the *afa* and *sfa* or the *pap* genes, corroborating two other studies [44,51]. This finding suggests that this strain may use other fimbrial or afimbrial adhesins to adhere to the urinary tract epithelium.

In this study, the two UPEC/aEPEC strains belonging to phylogenetic groups A and B1 presented a LAL pattern of adherence after 6 h of interaction with HeLa cells. Interestingly, our group´s previous study reported a UPEC strain that carried *eae* and was isolated in 1998 in the same hospital [36]. We have recently shown that such strain belongs to the phylogenetic group A, exhibits LAL, and produces actin aggregation in vitro, suggesting that it could promote AE lesions as aEPEC strains [52,53]. These characteristics may result from the epidemiological scenario added to the clones circulating in this specific geographic region when the study was carried out.

According to the criteria of intrinsic virulence proposed by Johnson et al. [16], five of the hybrid strains (HSP 60, HSP 93, HSP 117, HSP 215, and HSP 425) reported in the present study could be classified as ExPEC positive. This classification was based on the association of two or more genes that are more frequent in the strains; among them are *afa*, *iucD,* and *kpsMTII*, found in phylogroups A and D, unlike other studies that found this association to be more frequent in phylogroup B2 [54,55].

Spuberck et al. reported that *E. coli* strains carrying the *fyuA, chuA, yfcV*, and *vat* genes simultaneously could colonize the urinary tract more efficiently [19]. However, in the present study, only *fyuA* (seven strains) and *chuA* (one strain) were identified in the hybrid strains isolated from UTIs. Despite being criteria that predict the pathogenic potential of many strains, flaws in this classification may occur and they may not be applied to strains of extraintestinal origin or clinically significant strains [55,56,57,58]. Additionally, Spuberck et al. [19] showed that 70% of strains recovered from urine harbored these genetic markers, indicating that they are not universal among such strains, despite that they can cause UTIs. The bacterial genetic background might contribute to the absence of these traits. Moreover, although ExPEC strains have been recognized as commonly belonging to phylogroups B2 and D, most of the hybrid strains detected in our study belonged to phylogroup A, associated with DEC pathotypes. Regarding the pathogenic potential of these hybrid strains, the literature associates the presence of the *pap*, *sfa*, and *hly* genes to the phylogenetic B2 and D groups [59,60]; however, most of the hybrid strains (*n* = 8/9) of the present study belonged to the phylogenetic A and B1 groups, as previously reported [33,57,61]. These findings demonstrated that the phylogroups that often have commensal strains might also bear strains with high pathogenic potential.

Recurrent UPEC infections have been related to biofilm formation, which can determine the persistence of such pathogens in the vaginal microbiota, in the bladder epithelial cells, or both [62]. In addition, in patients using a bladder catheter, bacteria can ascend to the bladder and migrate to the mucosa and catheter surfaces, favoring infection [63]. In these cases, the pathogen’s ability to adhere and form biofilms on the device results in persistent and recurrent infections in catheterized patients [64,65]. It is also known that biofilm expression among EAEC strains is an essential determinant for the establishment of diarrheal disease. This process shows considerable complexity since it involves numerous adhesins and other non-adhesive factors [66]. Our findings corroborate some studies that also reported strong biofilm formation by UPEC strains in both LB and DMEM medium [67,68]. This ability contributes to the pathogen protection against host immunity and antimicrobials’ action, favoring the occurrence of persistent infections [69].

The VFs of the EAEC pathotype are regulated by the presence of the pAA plasmid and the main virulence transcriptional regulator *aggR* [70]. However, it must be taken into account that some fimbriae, such as AAF/I, curli, F9, type III fimbria, and other structures, are also related to biofilm production and virulence of EAEC strains [71]. Our group is further investigating the structures that might be involved in this process.

The production of ESβL by UPEC clones causing hospital- and community-acquired extraintestinal infections is the main concern since such pathogens are generally multi-drug resistant [72]. The production of CTX-M enzymes, which constitute the largest group among EβBLs spread worldwide, are commonly found in UPEC clones [73,74]. Our study identified the production of CTX-M-15, a variant widely distributed in Brazil and worldwide. A diversity of VFs combined with the production of ESβL in hybrid *E. coli* strains, such as the HSP 446 strain, could favor rapid colonization, persistence, and consequently dissemination of these strains that could potentially cause more severe diseases [75].

## 5. Conclusions

Our findings contribute to a better understanding of the occurrence and the pathogenic potential of hybrid *E. coli* strains, which can be related to severe cases of UTIs and intestinal/extraintestinal diseases. Furthermore, these findings present promising insights about the pathogenicity of hybrid strains that should be addressed to improve prevention and control measures.

## Figures and Tables

**Table 1:** Genetic markers of diarrheagenic *Escherichia coli* (DEC) pathotypes used in the classification of uropathogenic *E. coli* (UPEC) as a hybrid strain, and results obtained by virulence phenotypic tests.

Strain	Pathotype Genetic Markers			Hybrid Classification	Phylogroup	Adherence Pattern ^c^	Hemolysis ^d^	Biofilm Formation/Medium ^e^	
	DEC	ExPEC ^a^	UPEC ^b^					DMEM	LB
HSP 60	*aggR*	*iucD*, *kpsMTII*, *papC*	*fyuA*, *chuA*	UPEC/EAEC	D	NC	+ (*hlyA*)	+	+
HSP 93	*aggR*	*afaBCIII*, *iucD*, *kpsMTII*	*fyuA*	UPEC/EAEC	A	NC	+ (*hlyA*)	−	+
HSP 117	*aggR*	*afaBCIII*, *iucD*, *kpsMTII*	*fyuA*	UPEC/EAEC	A	NC	+ (*hlyA*)	+	-
HSP 199	*aggR*	-	-	UPEC/EAEC	B1	AA	_	−	+
HSP 215	*aggR*	*iucD*, *kpsMTII*	*fyuA*	UPEC/EAEC	A	NC	+ (*hlyA*)	+	+
HSP 414	*aggR*	-	*fyuA*	UPEC/EAEC	B1	AA	-	+	−
HSP 425	*aggR*	*afaBCIII, iucD*, *kpsMTII*, *papC*	*fyuA*	UPEC/EAEC	A	NC	+ (*hlyA*)	−	+
HSP 278	*eae*	*iucD*	*fyuA*	UPEC/aEPEC	A	LAL	−	−	−
HSP 446	*eae*	-	*-*	UPEC/aEPEC	B1	LAL	−	−	−

^a^ Presence of at least two among five (*pap*, *afa*/*dra*, *sfa*, *kpsMT*II, and *iut*/*iuc*) genes determine the intrinsic virulence of the strains. ^b^ Simultaneous presence of *vat*, *chuA*, *fyuA*, and *yfcV* determines the uropathogenic potential of the strains. ^c^ As determined in HeLa cells. NC: non-characteristic aggregative adherence pattern; AA: aggregative adherence; LAL: localized adherence-like. ^d^ Assessed after incubation for 3 h, 6 h, and 24 h on washed blood agar containing 10 mM CaCl_2;_ positive hemolytic strains detected in 3 h of incubation, except for HSP 425, which was detected after 6 h_;_ *hly+*: presence of the *hlyA* gene. ^e^ DMEM, Dulbecco Minimal Essential Medium; LB: Lysogeny Broth; (+) biofilm formation; (−): lack of biofilm formation.

**Table 2:** Pathotype, infection type ^a^, additional information, age and gender of the patients carrying hybrid *Escherichia coli* isolated from urinary tract infections.

Strain	Pathotype	Diagnosis	Additional Information	Age/Gender
HSP 60	UPEC/EAEC	Cystitis	None	85/female
HSP 93	UPEC/EAEC	Cystitis	None	26/female
HSP 117	UPEC/EAEC	Cystitis	None	40/female
HSP 199	UPEC/EAEC	Pyelonephritis	Recurrent UTIs ^c^	66/female
HSP 215	UPEC/EAEC	Cystitis	Recurrent UTIs ^c^	39/female
HSP 414	UPEC/EAEC	Cystitis	None	58/female
HSP 425	UPEC/EAEC	Unavailable ^b^	Chronic renal disease	61/male
HSP 278	UPEC/EPEC	Cystitis	None	22/female
HSP 446	UPEC/EPEC	Unavailable	Oncological inpatient	4/female

^a^ Types of urinary tract infection and the medical conditions were reported as they were described in the anonymized medical records. ^b^ The anonymized medical records of chronic renal inpatients and oncological inpatients were not available. ^c^ Urinary tract infections.

**Table 3:** Antimicrobial susceptibility profile and β-lactamase-encoding genes content in the nine hybrid uropathogenic *Escherichia coli* (UPEC) strains.

Strain	ESBL Gene	CAZ	FEP	CRO	ATM	MEM	ETP	IMI	APS	LEV	CIP	AMK	GEN	MIN	TGC	COL	PMB
HSP 60		0.5	1	0.5	0.125	<0.06	<0.06	0.5	**128/4**	0.06	0.125	**>256**	**8**	16	0.125	<0.25	<0.25
HSP 93		<0.125	<0.125	1	<0.06	<0.06	<0.06	<0.06	08/4	0.06	0.06	1	0.5	0.5	0.125	<0.25	<0.25
HSP 117		<0.125	<0.125	0.5	0.125	<0.06	<0.06	<0.06	02/4	0.5	**4**	8	0.5	8	0.25	<0.25	<0.25
HSP 199		0.5	0.25	1	<0.06	<0.06	<0.06	**<0.06**	**128/4**	**2**	**8**	**>256**	**4**	2	0.125	<0.25	<0.25
HSP 215		<0.125	<0.125	1	<0.06	<0.06	<0.06	<0.06	16/4	0.06	0.06	1	0.5	0.5	0.125	0.5	<0.25
HSP 425		<0.125	<0.125	**2**	1	<0.06	<0.06	<0.06	04/4	0.06	<0.03	1	1	1	0.125	<0.25	<0.25
HSP 278		<0.125	<0.125	0.5	0.125	<0.06	0.5	0.125	**128/4**	0.5	0.06	256	>256	8	0.125	<0.25	<0.25
HSP 446	*bla*_CTX-M-15_	**64**	**64**	**256**	**128**	<0.06	0.125	0.5	**128/4**	0.25	**1**	2	**8**	16	0.25	<0.25	<0.25

Abbreviations: AMK, amikacin; APS, ampicillin/sulbactam; ATM, aztreonam; CAZ, ceftazidime; CIP, ciprofloxacin; CRO, ceftriaxone; CST, colistin; ETP, ertapenem; FEP, cefepime; IPM, imipenem; GEN, gentamicin; MEM, meropenem; LEV, levofloxacin; MIN, minocycline; PMB, polymyxin B; TGC, tigecycline; COL, colistin. Numbers in bold represent the resistant phenotype according to BrCAST breakpoints.

## Data Availability

Not applicable.

## Supplementary Materials

The following are available online at https://www.mdpi.com/article/10.3390/microorganisms9040693/s1, Table S1: Virulence markers of DEC pathotypes investigated. Figure S1. Adherence pattern of hybrid uropathogenic *Escherichia coli* (UPEC) strains. Figure S2. Interaction with a renal origin cell-lineage.

## References

[1] Kaper J.B., Nataro J.P., Mobley H.L.T. (2004). Pathogenic *Escherichia coli*. Nat. Rev. Microbiol..

[2] Nataro J.P., Kaper J.B. (1998). Diarrheagenic *Escherichia coli*. Clin. Microbiol. Rev..

[3] Pitout J.D.D. (2012). Extraintestinal pathogenic *Escherichia coli*: A combination of virulence with antibiotic resistance. Front. Microbiol..

[4] Leimbach A., Hacker J., Dobrindt U. (2013). *E. coli* as an all-rounder: The thin line between commensalism and pathogenicity. Curr. Top. Microbiol. Immunol..

[5] Manges A.R., Geum H.M., Guo A., Edens T.J., Fibke C.D., Pitout J.D.D. (2019). Global extraintestinal pathogenic *Escherichia coli* (Expec) lineages. Clin. Microbiol. Rev..

[6] Ewers C., Li G., Wilking H., Kiebling S., Alt K., Antão E., Laturnus C., Diehl I., Glodde S., Homeier T. (2007). Avian pathogenic, uropathogenic, and newborn meningitis-causing *Escherichia coli*: How closely related are they?. Int. J. Med. Microbiol..

[7] Johnson J.R., Russo T.A. (2018). Molecular Epidemiology of Extraintestinal Pathogenic *Escherichia coli*. EcoSal Plus.

[8] Bien J., Sokolova O., Bozko P. (2012). Role of uropathogenic *Escherichia coli* virulence factors in development of urinary tract infection and kidney damage. Int. J. Nephrol..

[9] Ambrosi C., Sarshar M., Aprea M.R., Pompilio A., Di Bonaventura G., Strati F., Pronio A., Nicoletti M., Zagaglia C., Palamara A.T. (2019). Colonic adenoma-associated *Escherichia coli* express specific phenotypes. Microbes Infect..

[10] Campos S.C., Elkins J.M., Sheele J.M. (2021). Descriptive analysis of prostatitis in the emergency department. Am. J. Emerg. Med..

[11] Foxman B., Brown P. (2003). Epidemiology of urinary tract infections: Transmission and risk factors, incidence, and costs. Infect. Dis. Clin. N. Am..

[12] Sarshar M., Behzadi P., Ambrosi C., Zagaglia C., Palamara A.T., Scribano D. (2020). FimH and Anti-Adhesive Therapeutics: A Disarming Strategy Against Uropathogens. Antibiotics.

[13] Terlizzi M.E., Gribaudo G., Maffei M.E. (2017). UroPathogenic *Escherichia coli* (UPEC) infections: Virulence factors, bladder responses, antibiotic, and non-antibiotic antimicrobial strategies. Front. Microbiol..

[14] Yamaji R., Rubin J., Thys E., Friedman C.R., Riley L.W. (2018). Persistent pandemic lineages of uropathogenic *Escherichia coli* in a college community from 1999 to 2017. J. Clin. Microbiol..

[15] Begier E., Rosenthal N.A., Gurtman A., Kartashov A., Donald R.G.K., Lockhart S.P. (2021). Epidemiology of Invasive *Escherichia coli* Infection and Antibiotic Resistance Status Among Patients Treated in US Hospitals: 2009–2016. Clin. Infect. Dis..

[16] Johnson J.R., Murray A.C., Gajewski A., Sullivan M., Snippes P., Kuskowski M.A., Smith K.E. (2003). Isolation and molecular characterization of nalidixic acid-resistant extraintestinal pathogenic *Escherichia coli* from retail chicken products. Antimicrob. Agents Chemother..

[17] Johnson J.R., Kuskowski M., Denamur E., Elion J., Picard B. (2000). Clonal origin, virulence factors, and virulence (multiple letters). Infect. Immun..

[18] Picard B., Garcia J.S., Gouriou S., Duriez P., Brahimi N., Bingen E., Elion J., Denamur E. (1999). The link between phylogeny and virulence in *Escherichia coli* extraintestinal infection?. Infect. Immun..

[19] Spurbeck R.R., Dinh P.C., Walk S.T., Stapleton A.E., Hooton T.M., Nolan L.K., Kim K.S., Johnson J.R., Mobley H.L.T. (2012). *Escherichia coli* Isolates That Carry *vat*, *fyuA*, *chuA*, and *yfcV* Efficiently Colonize the Urinary Tract. Infect. Immun..

[20] Gomes T.A.T., Elias W.P., Scaletsky I.C.A., Guth B.E.C., Rodrigues J.F., Piazza R.M.F., Ferreira L.C.S., Martinez M.B. (2016). Diarrheagenic *Escherichia coli*. Braz. J. Microbiol..

[21] Lindstedt B.-A., Finton M.D., Porcellato D., Brandal L.T. (2018). High frequency of hybrid *Escherichia coli* strains with combined Intestinal Pathogenic *Escherichia coli* (IPEC) and Extraintestinal Pathogenic *Escherichia coli* (ExPEC) virulence factors isolated from human faecal samples. BMC Infect. Dis..

[22] Johnson J.R., Brown J.J. (1996). A novel multiply primed polymerase chain reaction assay for identification of variant *papG* genes encoding the Gal(α1-4)Gal-binding PapG adhesins of *Escherichia coli*. J. Infect. Dis..

[23] Clermont O., Christenson J.K., Denamur E., Gordon D.M. (2013). The Clermont *Escherichia coli* phylo-typing method revisited: Improvement of specificity and detection of new phylo-groups. Environ. Microbiol. Rep..

[24] Schmidt H., Beutin L., Karch H. (1995). Molecular analysis of the plasmid-encoded hemolysin of *Escherichia coli* O157:H7 strain EDL 933. Infect. Immun..

[25] Beutin L., Montenegro M.A., Orskov I., Orskov F., Prada J., Zimmermann S., Stephan R. (1989). Close association of verotoxin (shiga-like toxin) production with enterohemolysin production in strains of *Escherichia coli*. J. Clin. Microbiol..

[26] Rodrigues J., Scaletsky I.C.A., Campos L.C., Gomes T.A.T., Whittam T.S., Trabulsi L.R. (1996). Clonal structure and virulence factors in strains of *Escherichia coli* of the classic serogroup O55. Infect. Immun..

[27] Wakimoto N., Nishi J., Sheikh J., Nataro J.P., Sarantuya J., Iwashita M., Manago K., Tokuda K., Yoshinaga M., Kawano Y. (2004). Quantitative biofilm assay using a microtiter plate to screen for enteroaggregative *Escherichia coli*. Am. J. Trop. Med. Hyg..

[28] Bauer A.W., Kirby W.M.M., Sherris J.C., Turck M. (1996). Technical section. Am. J. Clin. Pathol..

[29] Nicoletti A.G., Marcondes M.F.M., Martins W.M.B.S., Almeida L.G.P., Nicolás M.F., Vasconcelos A.T.R., Oliveira V., Gales A.C. (2015). Characterization of BKC-1 Class A Carbapenemase from Klebsiella pneumoniae Clinical Isolates in Brazil. Antimicrob. Agents Chemother..

[30] Dobrindt U., Agerer F., Michaelis K., Janka A., Buchrieser C., Samuelson M., Svanborg C., Gottschalk G., Karch H., Hacker J. (2003). Analysis of genome plasticity in pathogenic and commensal *Escherichia coli* isolates by use of DNA arrays. J. Bacteriol..

[31] Dobrindt U. (2005). (Patho-)Genomics of *Escherichia coli*. Int. J. Med. Microbiol..

[32] Santos A.C.M., Santos F.F., Silva R.M., Gomes T.A.T. (2020). Diversity of Hybrid- and Hetero-Pathogenic *Escherichia coli* and their potential implication in more severe diseases. Front. Cell. Infect. Microbiol..

[33] Lara F.B.M., Nery D.R., de Oliveira P.M., Araujo M.L., Carvalho F.R.Q., Messias-Silva L.C.F., Ferreira L.B., Faria-Junior C., Pereira A.L. (2017). Virulence Markers and Phylogenetic Analysis of *Escherichia coli* Strains with Hybrid EAEC/UPEC Genotypes Recovered from Sporadic Cases of Extraintestinal Infections. Front. Microbiol..

[34] Toval F., Köhler C.D., Vogel U., Wagenlehner F., Mellmann A., Fruth A., Schmidt M.A., Karch H., Bielaszewska M., Dobrindt U. (2014). Characterization of *Escherichia coli* isolates from hospital inpatients or outpatients with urinary tract infection. J. Clin. Microbiol..

[35] Ogura Y., Ooka T., Asadulghani, Terajima J., Nougayrède J.P., Kurokawa K., Tashiro K., Tobe T., Nakayama K., Kuhara S. (2007). Extensive genomic diversity and selective conservation of virulence-determinants in enterohemorrhagic *Escherichia coli* strains of O157 and non-O157 serotypes. Genome Biol..

[36] Abe C.M., Salvador F.A., Falsetti I.N., Vieira M.A.M., Blanco J., Blanco J.E., Blanco M., Machado A.M.O., Elias W.P., Hernandes R.T. (2008). Uropathogenic *Escherichia coli* (UPEC) strains may carry virulence properties of diarrhoeagenic *E. coli*. FEMS Immunol. Med. Microbiol..

[37] Bielaszewska M., Schiller R., Lammers L., Bauwens A., Fruth A., Middendorf B., Schmidt M.A., Tarr P.I., Dobrindt U., Karch H. (2014). Heteropathogenic virulence and phylogeny reveal phased pathogenic metamorphosis in *Escherichia coli* O2: H6. EMBO Mol. Med..

[38] Boll E.J., Struve C., Boisen N., Olesen B., Stahlhut S.G., Krogfelt K.A. (2013). Role of enteroaggregative *Escherichia coli* virulence factors in uropathogenesis. Infect. Immun..

[39] Moraes C.T.P., Longo J., Silva L.B., Pimenta D.C., Carvalho E., Morone M.S.L.C., da Rós N., Serrano S.M.T., Santos A.C.M., Piazza R.M.F. (2020). Surface Protein Dispersin of Enteroaggregative *Escherichia coli* Binds Plasminogen That Is Converted Into Active Plasmin. Front. Microbiol..

[40] Galardini M., Clermont O., Baron A., Busby B., Dion S., Schubert S., Beltrao P., Denamur E. (2020). Major role of iron uptake systems in the intrinsic extra-intestinal virulence of the genus *Escherichia* revealed by a genome-wide association study. PLoS Genet..

[41] Yamamoto S. (2007). Molecular epidemiology of uropathogenic *Escherichia coli*. J. Infect. Chemother..

[42] Vieira M.A.M. (2009). Ilhas de patogenicidade. O Mundo da Saúde.

[43] Wiles T.J., Kulesus R.R., Mulvey M.A. (2008). Origins and virulence mechanisms of uropathogenic *Escherichia coli*. Exp. Mol. Pathol..

[44] Firoozeh F., Saffari M., Neamati F., Zibaei M. (2014). Detection of virulence genes in *Escherichia coli* isolated from patients with cystitis and pyelonephritis. Int. J. Infect. Dis..

[45] Nataro J.P., Yikang D., Cookson S., Cravioto A., Savarino S.J., Guers L.D., Levine M.M., Tacket C.O. (1995). Heterogeneity of enteroaggregative *Escherichia coli* virulence demonstrated. J. Infect. Dis..

[46] Gomes T.A.T., Abe C.M., Marques L.R.M. (1995). Detection of HeLa cell-detaching activity and alpha-hemolysin production in enteroaggregative *Escherichia coli* strains isolated from feces of Brazilian children. J. Clin. Microbiol..

[47] Marques L.R.M., Abe C.M., Griffin P.M., Gomes T.A.T. (1995). Association between alpha-hemolysin production and HeLa cell-detaching activity in fecal isolates of *Escherichia coli*. J. Clin. Microbiol..

[48] Yamamoto S., Terai A., Yuri K., Kurazono H., Takeda Y., Yoshida O. (1995). Detection of urovirulence factors in *Escherichia coli* by multiplex polymerase chain reaction. FEMS Immunol. Med. Microbiol..

[49] Terai A., Yamamoto S., Mitsumori K., Okada Y., Kurazono H., Takeda Y., Yoshida O. (1997). *Escherichia coli* Virulence Factors and Serotypesin Acute Bacterial Prostatitis. Int. J. Urol..

[50] Blanco M., Blanco J.E., Alonso M.P., Mora A., Balsalobre C., Muñoa F., Juárez A., Blanco J. (1997). Detection of pap, sfa and afa adhesin-encoding operons in uropathogenic *Escherichia coli* strains: Relationship with expression of adhesins and production of toxins. Res. Microbiol..

[51] Qin X., Hu F., Wu S., Ye X., Zhu D., Zhang Y., Wang M. (2013). Comparison of Adhesin Genes and Antimicrobial Susceptibilities between Uropathogenic and Intestinal Commensal *Escherichia coli* Strains. PLoS ONE.

[52] Valiatti T.B., Santos F.F., Santos A.C.M., Silva R.M., Carvalho E., Gomes T.A.T. (2019). Draft Whole-Genome Sequence of a Uropathogenic *Escherichia coli* Strain Carrying the eae Gene. Microbiol. Resour. Announc..

[53] Valiatti T.B., Santos F.F., Santos A.C.M., Nascimento J.A.S., Silva R.M., Carvalho E., Sinigaglia R., Gomes T.A.T. (2020). Genetic and Virulence Characteristics of a Hybrid Atypical Enteropathogenic and Uropathogenic *Escherichia coli* (aEPEC/UPEC) Strain. Front. Cell. Infect. Microbiol..

[54] Johnson J.R., Kuskowski M.A., Menard M., Gajewski A., Xercavins M., Garau J. (2006). Similarity between human and chicken *Escherichia coli* isolates in relation to ciprofloxacin resistance status. J. Infect. Dis..

[55] Freire C.A., Santos A.C.M., Pignatari A.C., Silva R.M., Elias W.P. (2020). Serine protease autotransporters of Enterobacteriaceae (SPATEs) are largely distributed among *Escherichia coli* isolated from the bloodstream. Braz. J. Microbiol..

[56] Santos A.C.M., Zidko A.C.M., Pignatari A.C., Silva R.M. (2013). Assessing the diversity of the virulence potential of *Escherichia coli* isolated from bacteremia in São Paulo, Brazil. Braz. J. Med. Biol. Res..

[57] Olesen B., Scheutz F., Andersen R.L., Menard M., Boisen N., Johnston B., Hansen D.S., Krogfelt K.A., Nataro J.P., Johnson J.R. (2012). Enteroaggregative *Escherichia coli* O78:H10, the cause of an outbreak of urinary tract infection. J. Clin. Microbiol..

[58] Campos A.C.C., Andrade N.L., Ferdous M., Chlebowicz M.A., Santos C.C., Correal J.C.D., Lo Ten Foe J.R., Rosa A.C.P., Damasco P.V., Friedrich A.W. (2018). Comprehensive Molecular Characterization of *Escherichia coli* Isolates from Urine Samples of Hospitalized Patients in Rio de Janeiro, Brazil. Front. Microbiol..

[59] Johnson J.R., Delavari P., Kuskowski M., Stell A.L. (2001). Phylogenetic Distribution of Extraintestinal Virulence-Associated Traits in *Escherichia coli*. J. Infect. Dis..

[60] Lee J.H., Subhadra B., Son Y.J., Kim D.H., Park H.S., Kim J.M., Koo S.H., Oh M.H., Kim H.J., Choi C.H. (2016). Phylogenetic group distributions, virulence factors and antimicrobial resistance properties of uropathogenic *Escherichia coli* strains isolated from patients with urinary tract infections in South Korea. Lett. Appl. Microbiol..

[61] Dias R.C.S., Marangoni D.V., Smith S.P., Alves E.M., Pellegrino F.L.P.C., Riley L.W., Moreira B.M. (2009). Clonal composition of *Escherichia coli* causing community-acquired urinary tract infections in the state of Rio de Janeiro, Brazil. Microb. Drug Resist..

[62] Soto S.M. (2014). Importance of Biofilms in Urinary Tract Infections: New Therapeutic Approaches. Adv. Biol..

[63] Narayanan A., Nair M.S., Muyyarikkandy M.S., Amalaradjou M.A. (2018). Inhibition and inactivation of uropathogenic *Escherichia coli* Biofilms on urinary catheters by Sodium Selenite. Int. J. Mol. Sci..

[64] Nicolle L.E. (2014). Catheter associated urinary tract infections. Antimicrob. Resist. Infect. Control.

[65] Eberly A.R., Floyd K.A., Beebout C.J., Colling S.J., Fitzgerald M.J., Stratton C.W., Schmitz J.E., Hadjifrangiskou M. (2017). Biofilm formation by uropathogenic *Escherichia coli* is favored under oxygen conditions that mimic the bladder environment. Int. J. Mol. Sci..

[66] Pereira A.L., Silva T.N., Gomes A.C., Arajo A.C. (2010). Diarrhea-associated biofilm formed by enteroaggregative *Escherichia coli* and aggregative *Citrobacter freundii*: A consortium mediated by putative F pili. BMC Microbiol..

[67] Zamani H., Salehzadeh A. (2018). Biofilm formation in uropathogenic *Escherichia coli*: Association with adhesion factor genes. Turk. J. Med. Sci..

[68] Shah C., Baral R., Bartaula B., Shrestha L.B. (2019). Virulence factors of uropathogenic *Escherichia coli* (UPEC) and correlation with antimicrobial resistance. BMC Microbiol..

[69] Soto S.M., Smithson A., Horcajada J.P., Martinez J.A., Mensa J.P., Vila J. (2006). Implication of biofilm formation in the persistence of urinary tract infection caused by uropathogenic *Escherichia coli*. Eur. Soc. Clin. Infect. Dis..

[70] Baudry B., Savarino S.J., Vial P., Kaper J.B., Levine M.M. (1990). A sensitive and specific dna probe to identify enteroaggregative *Escherichia coli*, a recently discovered diarrheal pathogen. J. Infect. Dis..

[71] Lüthje P., Brauner A. (2014). Virulence Factors of Uropathogenic *E. coli* and Their Interaction with the Host. Adv. Microb. Physiol..

[72] Wollheim C., Guerra I.M.F., Conte V.D., Hofman S.P., Schreiner F.J., Delamare A.P.L., Barth A.L., Echeverrigaray S., Da Costa S.O.P. (2011). Nosocomial and community infections due to class A extended-spectrum β-lactamase (ESBlA)-producing *Escherichia coli* and Klebsiella spp. in southern Brazil. Braz. J. Infect. Dis..

[73] Gonçalves L.F., de Oliveira Martins-Júnior P., de Melo A.B.F., da Silva R.C.R.M., de Paulo Martins V., Pitondo-Silva A., de Campos T.A. (2016). Multidrug resistance dissemination by extended-spectrum β-lactamase-producing *Escherichia coli* causing community-acquired urinary tract infection in the Central-Western Region, Brazil. J. Glob. Antimicrob. Resist..

[74] Bevan E.R., Jones A.M., Hawkey P.M. (2017). Global epidemiology of CTX-M β-lactamases: Temporal and geographical shifts in genotype. J. Antimicrob. Chemother..

[75] Subashchandrabose S., Mobley H.L.T. (2015). Virulence and Fitness Determinants of Uropathogenic *Escherichia coli*. Microbiol. Spectr..

