# Supplementary material for: Frequency and Diversity of Hybrid Escherichia coli Strains Isolated from Urinary Tract Infections
Source: Microorganisms. 2021 Mar 27;9(4):693. doi: 10.3390/microorganisms9040693 (PMC8065829; doi:10.3390/microorganisms9040693)
Supplement: Supplementary file 1 [file microorganisms-09-00693-s001.pdf]

**Table S1.** Virulence markers of diarrheagenic *Escherichia coli* (DEC) pathotypes investigated.

| DEC Pathotype[a] | Diagnostic markers | References |
|---|---|---|
| **typical EPEC** | *eae*, *bfpB* | [1,2] |
| **atypical EPEC** | *eae* | [1] |
| **STEC** | *stx* | [3] |
| **EAEC** | *aggR* and aggregative adherence pattern | [4] |
| **EIEC** | *invE* | [2] |
| **ETEC** | *elt*, *est* | [5] |

[a], EPEC, enteropathogenic *Escherichia coli*; STEC, Shiga-toxin producing *E. coli*; EAEC, enteroaggregative *E. coli*; EIEC, enteroinvasive *E. coli*; ETEC, enterotoxigenic *E. coli*.

**References**


1. Gannon, V.P.J.; Rashed, M.; King, R.K.; Thomas, E.J.G. Detection and Characterization of the eae Gene of Shiga-Like Toxin-Producing *Escherichia coli* Using Polymerase Chain Reaction. **1993**, *31*, 1268–1274.
2. Müller, D.; Hagedorn, P.; Brast, S.; Heusipp, G.; Bielaszewska, M.; Friedrich, A.W.; Karch, H.; Schmidt, M.A. Rapid Identification and Differentiation of Clinical Isolates of Enteropathogenic *Escherichia coli* (EPEC ), Atypical EPEC , and Shiga Toxin-Producing *Escherichia coli* by a One-Step Multiplex PCR Method. **2006**, *44*, 2626–2629, doi:10.1128/JCM.00895-06.
3. Cebula, T.A.; Payne, W.L.; Feng, P. Simultaneous Identification of Strains of Escherichia coli Serotype O157:H7 and Their Shiga-Like Toxin Type by Mismatch Amplification Mutation Assay-Multiplex PCR. **1995**, *33*, 248–250.
4. Andrade, F.B.; Gomes, T.A.T.; Elias, W.P. A sensitive and specific molecular tool for detection of both typical and atypical enteroaggregative *Escherichia coli*. *J. Microbiol. Methods* **2014**, *106*, 16–18, doi:10.1016/j.mimet.2014.07.030.
5. Stacy-phipps, S.; Mecca, J.J.; Weiss, J.B. Multiplex PCR Assay and Simple Preparation Method for Stool Specimens Detect Enterotoxigenic *Escherichia coli* DNA during Course of Infection. **1995**, *33*, 1054–1059.

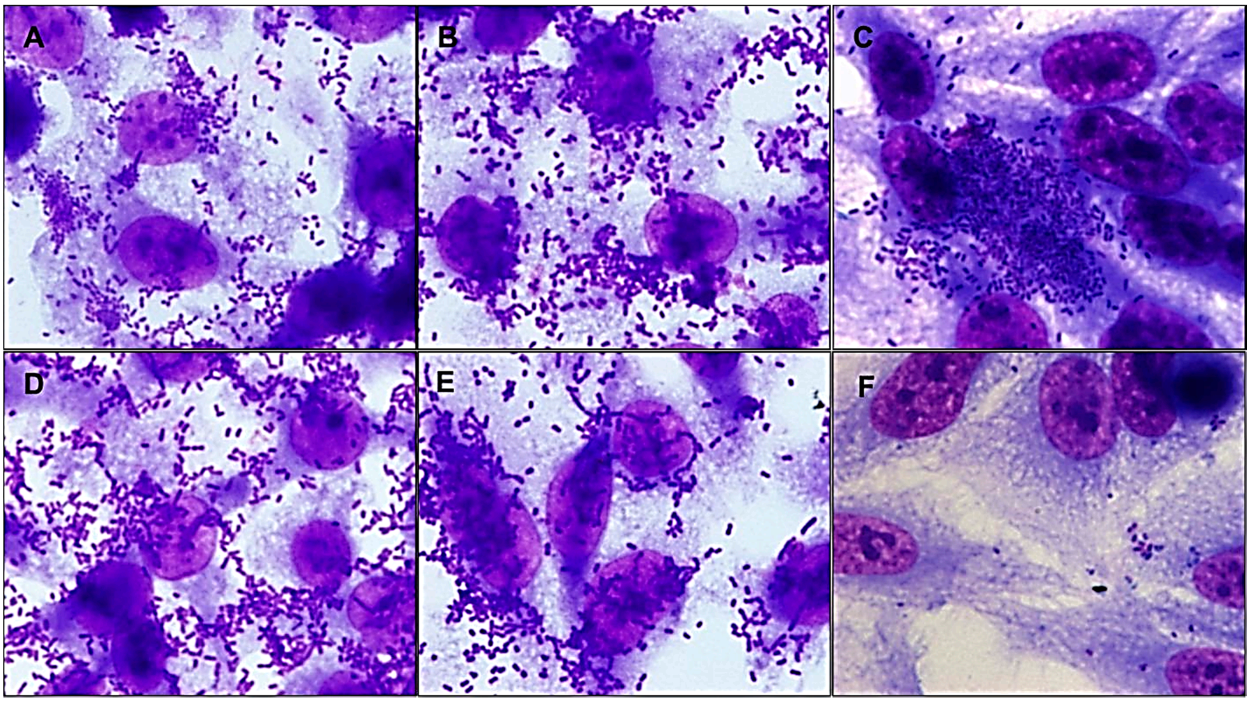


Figure S1. Adherence pattern of hybrid uropathogenic *Escherichia coli* (UPEC) strains. The adherence patterns were assessed as preconized in HeLa cells in assays with an incubation period of 3 h or 6 h, at 37 °C in the presence of 2% D-mannose, using a multiplicity of infection of 10. Preparations were stained with May-Grünwald/Giemsa and observed under a light optical microscope (1,000 x magnification). Hybrid UPEC/EAEC (enteroaggregative *E. coli*) strains are in panels **A**, **B**, **C**, **D**, and **E**, and a hybrid UPEC/aEPEC (atypical enteropathogenic *E. coli*) strain in panel **F.** All hybrid UPEC strains were adherent, and different adherence patterns were identified; the aggregative adherence pattern is observed in **C,** and the localized adherence-like pattern in **F**; strains in panels **A**, **B**, **D, E**, and **G** displayed a non-characteristic aggregative adherence (NC) pattern with small loose clusters and spread foci of adherent bacteria. **A.** HSP 60; **B.** HSP 93; **C.** HSP 199; **D.** HSP 215; **E.** HSP 425; **F.** HSP 446. The controls (not shown) were the same as those displayed in Figure 2 of the manuscript.

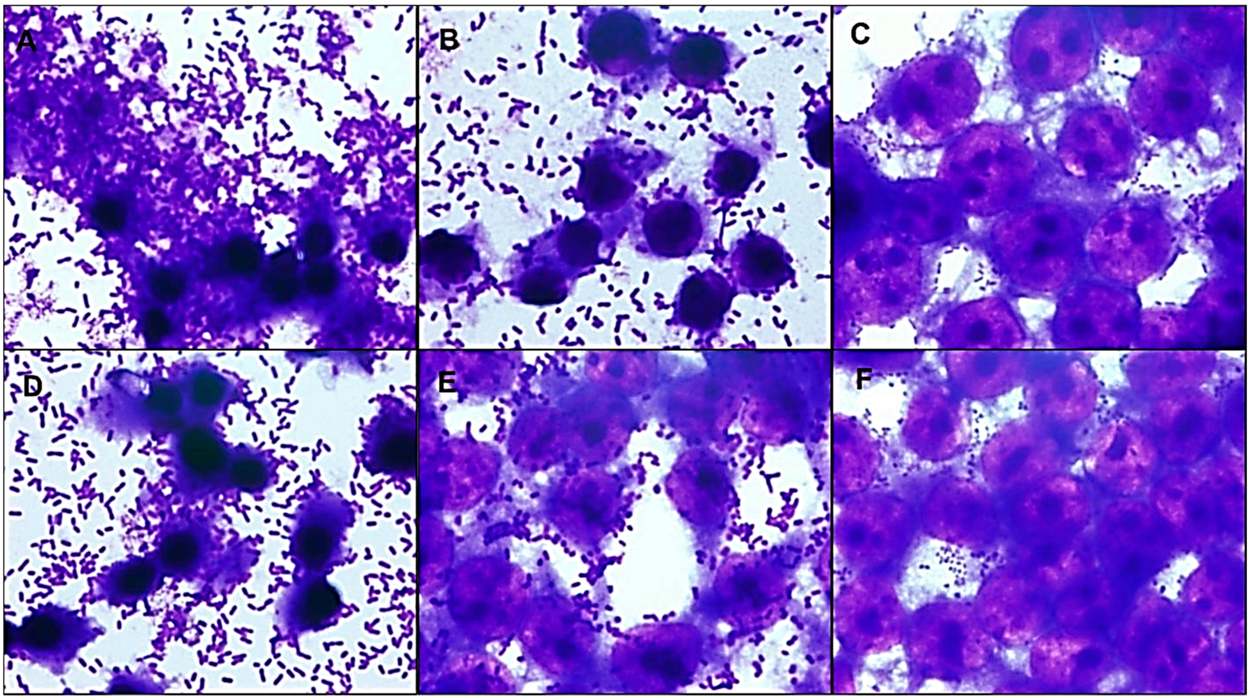


Figure S2. Interaction with a renal origin cell-lineage. The hybrid uropathogenic *Escherichia coli* (UPEC) strains'

capacity to interact with human renal cells was assessed using HEK 293T cells in assays with an incubation period of 3 h, at 37 °C without D-mannose, using a multiplicity of infection of 10. Preparations were stained with May-Grünwald/Giemsa and observed under a light optical microscope (1,000 x magnification). Hybrid UPEC/EAEC (enteroaggregative *E. coli*) strains are in panels **A**, **B**, **C**, **D**, and **E**, and a hybrid UPEC/aEPEC (atypical enteropathogenic *E. coli*) strain in panel **F**. All hybrid UPEC strains were capable of interacting with renal cells in diverse intensity; in panels **A**, **B,** and **D**, the HEK 293T cell monolayer was partially detached, and pyknotic nuclei are observed in the remaining cells. **A.** HSP 60; **B.** HSP 93; **C.** HSP 199; **D.** HSP 215; **E.** HSP 425; **F.** HSP 446. The controls (not shown) were the same as those displayed in Figure 3 of the manuscript.
